# Knowledge, Awareness, and Attitudes Toward Obstructive Sleep Apnea among the Population of the Asir Region of Saudi Arabia in 2019

**DOI:** 10.7759/cureus.7254

**Published:** 2020-03-13

**Authors:** Ali M Alshehri, Mohammed S Alshehri, Omar M Alamri, Fayez S Alshehri, Mazen Alshahrani, Mohammed A Alflan, Meshary S Alshahrani

**Affiliations:** 1 Medicine, King Khalid University, Al-Namas, SAU; 2 Medicine, King Khalid University, Khamis Mushait, SAU; 3 Medicine, King Khalid University, Abha, SAU; 4 Internal Medicine, Armed Forces Hospitals Southern Region, Khamis Mushait, SAU

**Keywords:** knowledge, awareness, obstructive sleep apnea, asir region, saudi arabia

## Abstract

Background

Obstructive sleep apnea (OSA) is a sleep disorder that involves the cessation or significant decrease in airflow along with a distinct effort to breathe. While there are several types of sleep apnea, OSA is the most common.

Methodology

This descriptive online cross-sectional study assessed knowledge, awareness, and attitudes towards OSA over 30 days in a nonrandomized sample of the general population of the Asir region of Saudi Arabia. Subjects were included if they lived in the Asir region and were aged >18 years.

Results

Of the respondents who participated in the study, 64% were aware of OSA, whereas 36% were not. Most respondents reported that OSA was dangerous, whereas 24% did not know whether OSA was dangerous. Moreover, 81% of respondents reported that they did not know about methods of diagnosing OSA, and 84% did not know about the methods to treat OSA.

Conclusions

Subjects living in the Asir region of Saudi Arabia showed a low level of awareness of all aspects of OSA. Their lack of sources of knowledge indicates the need for medical staff to improve awareness and knowledge of OSA.

## Introduction

Obstructive sleep apnea (OSA), the most common type of sleep apnea, is characterized by repeated episodes of complete or partial obstruction of the upper airways during sleep, despite efforts to breathe, and is associated with a reduction in blood oxygen saturation. OSA associated with excessive daytime sleepiness is commonly called OSA syndrome or OSA-hypopnea syndrome [1]. The recurrent episodes of upper airway collapse during sleep in subjects with OSA are associated with recurrent oxyhemoglobin desaturation and arousals from sleep [1].

Signs and symptoms of OSA include unexplained daytime sleepiness, restless sleep, and loud snoring (with periods of silence followed by gasps). Less common symptoms are morning headaches; insomnia; trouble concentrating; mood changes such as irritability, anxiety, and depression; forgetfulness; increased heart rate and/or blood pressure; decreased sex drive; unexplained weight gain; and increased urination and/or nocturia, and heavy night sweats. Awakenings in subjects with OSA are usually too brief for subjects to remember. Awakenings are frequently accompanied by shortness of breath, which corrects itself quickly within one or two deep breaths. Subjects may also make snorting, choking, or gasping sounds. These patterns can repeat five to more than 30 times per hour throughout the entire night [2]. These disruptions may impair subjects’ abilities to reach the desired deep, restful phases of sleep, with subjects likely feeling sleepy during waking hours. Subjects with OSA may be unaware that their sleep was interrupted, with many of these subjects thinking that they slept well throughout the night [3].

Diagnosis and treatment of OSA depend on subjects’ knowledge, awareness, and attitudes toward this condition. The present study, therefore, evaluated the knowledge, awareness, and attitudes toward OSA in persons living in the Asir region of Saudi Arabia. This study also assessed risk factors associated with OSA in this population, including obesity, hypertension, smoking, and family history; the main sources of information from which the public obtains information about sleep and how to improve it; and attitudes toward OSA, including its complications and awareness of the seriousness of these complications.

## Materials and methods

This self-funded descriptive online cross-sectional study involved a nonrandomized sample of the general population of the Asir region of Saudi Arabia. Questionnaires were administered online over 30 days to subjects living in the Asir region who were aged >18 years. The validated questionnaire survey was sent to social media sites used by the Saudi population, including Twitter, Facebook, WhatsApp, Telegram, and Snapchat, and conducted online via Google survey. The questionnaire included demographic aspects such as age, gender, and level of education, as well as several health-related characteristics, such as health status, body mass index, physical activity, and smoking.

The raw data were recorded on appropriate designed excel spreadsheets and processed in accordance with the best practices for raw data management to identify any inaccuracies prior to statistical analysis. Outlying data were flagged. Categorical variables were treated similarly to identify any potential anomalies. All identified anomalies were discussed with the biostatistics team and were corrected prior to initial statistical analysis. Descriptive statistics were recorded as numbers, percentages, means, and standard deviations.

The study protocol was approved by the ethics review board of our institution, and all participants provided written informed consent. Subjects were informed of their right to withdraw from the study at any time without any obligation toward the study team. The data were collected and used for research purposes only, with only the principal investigator having access to the data. Confidentiality was maintained throughout the study by keeping the participants’ identity anonymous, with the names and personal information of participants not obtained for ethical reasons.

## Results

The study sample consisted of 626 participants. Of these, 37% were aged ≥45 years, 27% were aged 36-45 years, 20% were aged 26-35 years, and 16% were aged 18-25 years (Table 1). Slightly less than two-thirds of respondents were males (61%), while 39% of participants were females. Analysis of respondent education level showed that most subjects were highly educated, with 64% having a bachelor`s degree, 6% having a master’s degree, and 6% having a Ph.D. In addition, 20% of participants had completed secondary school, whereas only 2% completed intermediate schools, and 1% completed primary schools (Table 2). Moreover, 28% of respondents worked in the healthcare field, whereas 72% did not.

**Table 1:** Age distribution of the participants in the study

	Number	Percent
18-25	102	16.3
26-35	124	19.8
36-45	171	27.3
45+	229	36.6
Total	626	100.0%

**Table 2:** Educational level of respondents

	Number	Percent
Primary	6	1.0
Intermediate	15	2.4
Secondary	124	19.8
University (Bachelor)	401	64.1
Master	40	6.4
PhD	40	6.4
Total	626	100.0%

The most frequent chronic diseases in our study respondents were diabetes mellitus (12%), hypertension (10%), vision problems (9%), and arthritis (7%), with 6% having other chronic diseases (Table 3). Most respondents (62%) were in good health, reporting no chronic diseases.

**Table 3:** Percentages of participants with chronic diseases

	Number	Percent
Diabetes mellitus	73	11.7
Hypertension	60	9.6
High cholesterol	2	0.3
Depression	14	2.2
Heart disease	15	2.4
Arthritis	45	7.2
Vision problems	57	9.1
Respiratory diseases	30	4.8
Cancer	1	0.2
Others	39	6.2
None	389	62.1
Total	626	

Most subjects (54%) reported sleeping for four to six hours per day, and 41% reported sleeping for seven to nine hours per day. Only 3% reported sleeping for more than nine hours per day, whereas 2% reported sleeping for three or fewer hours per day. Assessments of participants’ knowledge about the number of hours of sleep needed per day showed that the majority (82%) regarded that sleep for seven to nine hours was needed, with 14% reporting that four to six hours per day were needed (Table 4). Only 4% reported that sleep for more than nine hours per day was needed, and only 0.2% reported that with three or fewer hours were required. About 70% of the respondents reported that the time they slept was sufficient for performing daily activities without difficulties, whereas 30% did not. Assessment of participants’ knowledge of the most important benefits of adequate sleep found that 51% regarded physical benefits, 41% enhanced thinking, 5% reported emotional benefits, and 3% regarded social benefits as most important.

**Table 4:** Participants’ knowledge of sleep and need to sleep

Hours of sleep needed per day		
Time (hours)	Number	Percent
≤3	1	0.2
4-6	87	13.9
7-9	514	82.1
9+	24	3.8
Sleep time is sufficient for performing daily activities without difficulties		
Yes	433	69.2
No	193	30.8
Most important benefits of adequate sleep		
Physical benefits	317	50.6
Enhanced thinking	259	41.4
Social benefits	16	2.6
Emotional benefits	34	5.4
Lack of sleep affects work		
Yes	611	97.6
No	15	2.4
Drowsiness during the day		
Yes	375	59.9
No	251	40.1
Nap during the day		
Yes	468	74.8
No	158	25.2
Nap during the day affects sleep at night		
Yes	319	51.0
No	307	49.0
Relationship between sleep and aging		
Yes	444	70.9
No	45	7.2
Do not know	137	21.9
Total	626	100.0

Almost all respondents (98%) believed that lack of sleep affected their work, whereas 60% reported drowsiness during the day. Although 75% reported taking a nap during the day, 51% were aware that naps during the day affect sleep at night. In addition, 71% of respondents reposted knowledge of a relationship between sleep and aging, whereas 22% were unaware of this relationship.

Of the study participants, 64% had heard of OSA, whereas 36% had not. About 75% regarded OSA as dangerous, whereas 24% did not know whether it is dangerous or not. Moreover, 81% of respondents reported never hearing about methods of diagnosing OSA, with 84% being unaware of methods used to treat OSA. The majority of respondents (96%) had never been diagnosed with OSA, with 80% regarding the diagnosis and treatment of sleep apnea as important.

## Discussion

This cross-sectional study was undertaken to determine the knowledge, awareness, and attitudes toward OSA among the general population of the Asir region of Saudi Arabia. Investigations of the presence or absence of risk factors for OSA (e.g., obesity, hypertension, smoking, family history), the main sources of information to the public about sleep and how to improve it, general attitudes toward OSA and its complications, and public awareness of the seriousness of these complications may help in the diagnosis and treatment of OSA.

Many studies worldwide have assessed awareness and knowledge of OSA, with one study in China reporting that 21.5% of the respondents were aware of OSA. A total of 77 (5.9%), 158 (12.1%), 150 (11.5%), and 110 (8.4%) respondents were able to correctly list at least one risk factor, symptom, health consequence, and treatment options for OSA, respectively [4]. Among the study participants of the Asir region of Saudi Arabia, 64% had heard of OSA; 93%, 89%, 73%, and 16% answers were correct regarding risk factors, symptoms, health consequence, and treatment options for OSA; the population education level of the first research showed 9.3% with no former education, 36.4% with secondary and below education, and 54.2% with post-secondary education. However, in this research, the education level showed secondary and post-secondary more than 95%.

The reason for the population to seek healthcare services is their understanding and knowledge regarding the symptoms and the complications; a study involving the general population in the Lorraine region of France showed despite encouraging results regarding OSA symptoms, the general population showed limited awareness of its complications. Innovative educational campaigns must be organized to inform practitioners and the general public about the disease and raise awareness of its complications [5]. A study in the USA found that OSA is a serious illness affecting about 12% of adults, with most of these patients being undiagnosed, posing serious healthcare and economic burdens. The STOP-BANG score (Snoring, Tiredness, Observed apnea, blood Pressure, Body mass index, Age, Neck circumference, and Gender) was reported to be a reliable screening tool to identify patients with probable OSA [6]. Early diagnosis by in-lab polysomnography or home sleep apnea test should be followed by an appropriate therapeutic modality. Currently, continuous positive airway pressure therapy remains the treatment of choice for patients with moderate and severe OSA.

The importance of lifestyle modifications such as smoking cessation, weight reduction, and physical activity found to be a way of treatment. Controlled intervention trials strongly suggest that weight reduction together with a healthy diet and increased physical activity may correct or at least improve the symptoms of OSA [7].

Knowledge sources about OSA are very important. In this study, we found (26%) of respondents seeking the doctors for information, while (23%) they seek it from social media. Research done about the quality of YouTube as a source of information showed that YouTube is a promising source of information for OSA patients. Educational and news videos are of the highest quality. General quality measures like search position, views, and likes are not correlated with formally scored value. Sleep surgery and otolaryngologists are minimally mentioned, representing an opportunity for improvement [8].

## Conclusions

The present study showed a poor level of awareness regarding all aspects of OSA among the general population of the Asir region of Saudi Arabia. Most subjects lacked sources of knowledge about this condition, indicating the need for medical personnel to improve awareness. Health education campaigns about OSA and its importance are needed, as is the training of primary healthcare physicians to raise awareness in their communities. Additional studies are also needed to assess the level of community awareness about OSA and its complications.
